# Editorial: Exploring molecular mechanisms and novel diagnostics in cardiovascular disease treatment

**DOI:** 10.3389/fcvm.2026.1792721

**Published:** 2026-03-10

**Authors:** Jianguo Sun, Yiwei Liu, Qianman Peng

**Affiliations:** 1 China Pharmaceutical University, Nanjing, China; 2 St Jude Children's Research Hospital, Memphis, TX, United States; 3 Houston Methodist Hospital, Houston, TX, United States; 4 Harvard Medical School, Boston, MA, United States; 5 Boston Children's Research, Boston, MA, United States

**Keywords:** novel therapeutics, cardiovascular pharmacology, cardiovascular disease, drug action, statistical modeling

**Editorial on the Research Topic**
Exploring molecular mechanisms and novel diagnostics in cardiovascular disease treatment

Cardiovascular diseases (CVDs) are the leading causes of mortality and morbidity worldwide. Pharmacological therapies have made substantial strides in managing these diseases through the development of various drugs, including antiplatelet agents (1), anticoagulants (2), cholesterol-lowering medications (3), and antihypertensives (4). Despite these advancements, these medications often come with adverse effects such as bleeding (5), skin necrosis (6), hypotension (7), dizziness (8), and arrhythmia (9). Thus, there remains an urgent need for the development of more effective and safer therapeutic options. Recent studies have explored diverse mechanisms of drug action, cell signaling pathways, and the roles of different cell types in cardiovascular diseases. However, gaps persist in our understanding of the pathogenesis, diagnosis, and treatment of CVDs, necessitating further research to address these unmet medical needs.

This topic particularly focuses on three different areas in drug action of new pharmacological interventions: (i) new understandings of cardiovascular diseases; (ii) novel insight of drug discovery and development; and (iii) data science and analytics. For this Research topic, we collected 7 original research papers, 2 clinical trial article and 6 review articles in the field of cardiovascular disease.

## New understandings of cardiomyopathy and vascular diseases

Firstly, we collected articles that provide novel understandings of cardiomyopathy and vascular diseases. Xu et al. provided an overview of the pathogenesis, evaluation, and treatment of hypertrophic cardiomyopathy (HCM) (Xu et al.). The primary purposes of current pharmacological interventions are to reduce clinical symptoms and improve quality of life in patients with obstructive HCM. Pharmacotherapy of patients with obstructive HCM, including traditional drugs (β-blockers; Verapamil), myosin inhibitors, and invasive treatments of obstructive HCM. Kim et al. from Stanford University published a clinical report on a 1-year experience with mavacamten and its effects on obstructive hypertrophic cardiomyopathy. Their data concluded that mavacamten, as a first-in-class cardiac myosin inhibitor, is efficacious and safe. The researchers found that mavacamten results in significant improvements in wall thickness and mitral regurgitation, as well as symptomatic improvement in patients. They also found that adverse events were rare (Kim et al.).

Qi et al.’s research on glucagon-like peptide receptor agonist GABABR activation counteracts glucagon-like peptide-1 receptor agonists chronotropic effects while synergistically enhancing anti-arrhythmic efficacy post-MI, which indicated a novel interaction in cardiac electrophysiology (Qi et al.). Wang et al. explored the effects of folic acid and vitamin B12, in combination with rosuvastatin, in treating coronary heart disease complicated by hyperlipidemia (Wang et al.). They report that this combination effectively improves cardiac function, blood lipids, and inflammatory responses, and is safe for clinical use in patients with coronary heart disease. One original research study showed that astragali radix-fescurainiae semen is effective in heart failure and presented the mechanisms underlying its efficacy, providing new insights into the clinical treatment of heart failure (Wang et al.). Another review provided a systematic analysis of the role of non-coding RNAs (ncRNAs) in modulating these pathological processes in autophagy and myocardial ischemia-reperfusion injury (Song et al.).

## The therapeutics development in cardiovascular diseases

In this collection, both original research articles and review papers provide insight into drug discovery and development in cardiovascular diseases. Zhai et al. introduced that finerenone is a third-generation non-steroidal mineralocorticoid receptor antagonist (Zhai et al.). Finerenone has been introduced to address knowledge gaps and to enable a broader evaluation of its clinical value, potentially informing existing treatment strategies. Chen et al. introduced the research progress of sea buckthorn (*Hippophae rhamnoides* L.) in the prevention and treatment of cardiovascular disease in a review article (Chen et al.).

One review article summarized that Chinese medicine targets cellular autophagy against cardiovascular diseases: atherosclerosis, diabetic cardiomyopathy; myocardial ischemia-reperfusion injury, and heart failure (Chen et al.). Huang et al. summarized the mechanism of action and potential therapeutic targets of the TGF-β related signaling pathway and its downstream miRNA expression in pulmonary arterial hypertension (Huang et al.). Wang et al. argue that preclinical research and clinical trials support miRNA-targeted therapy as an important approach for treating pulmonary arterial hypertension. They found that multigene-guided antiplatelet therapy is more effective in reducing adverse cardiovascular events in their research (Wang et al.). These findings indicated that traditional drugs, gene therapy, and other advanced therapies are used in cardiovascular drug discovery and development.

## Data science and analytics

In this area, we collected four articles that discussed HIV, hypertension, ischemic stroke and fibrosis in this theme collection. Yang et al. analyzed data for HIV infection and pulmonary arterial hypertension (PAH) from gene expression omnibus (GEO) database. It showed that CC-type chemokine ligand 5 (CCL5) might be a biomarker of both HIV infection and PAH and provided a new drug target for HIV associated PAH (Yang et al.). Another comprehensive search involving 767 participants and efficacy and safety evaluation of allisartan isoproxil in patients with hypertension, and benefits are achieved with minimal adverse reactions (Zhao et al.). Liu et al. applied animal research models, Kyoto Encyclopedia of Genes and Genomes (KEGG), and Gene Ontology (GO), and enrichment analysis on the intersection targets, and LVFP significantly inhibited myocardial fibrosis (Liu et al.). Nine identical genes (AKT1, SRC, HSP90AA1, GSK3β, ENO1, VEGFR2, RHOA, IL-2, and PKM) play roles in MF treatment by participating in signaling pathways related to multiple cardiovascular diseases. *Ligustrum vicaryi* L. fruit polysaccharide shows therapeutic potential for MF, primarily through the regulation of targets and various signaling pathways. Yu et al. performed a genetic association study for ischemic stroke. Genetic variants in glucagon-like peptide-1 receptor agonists targets exhibited a positive correlation with ischemic stroke risk, while genetic variation in dipeptidyl peptidase 4 inhibitors (DPP-4i) targets showed a negative association with ischemic stroke risk (Yu et al.).

In summary, the broad coverage of this research topic opens numerous new pharmacological perspectives on molecular mechanisms, evaluation, and therapeutic approaches in cardiovascular diseases. Continued research in these areas is crucial for translating emerging knowledge into meaningful clinical outcomes. Such an interdisciplinary approach could rapidly advance our understanding of the disease and help drug discovery and development.
